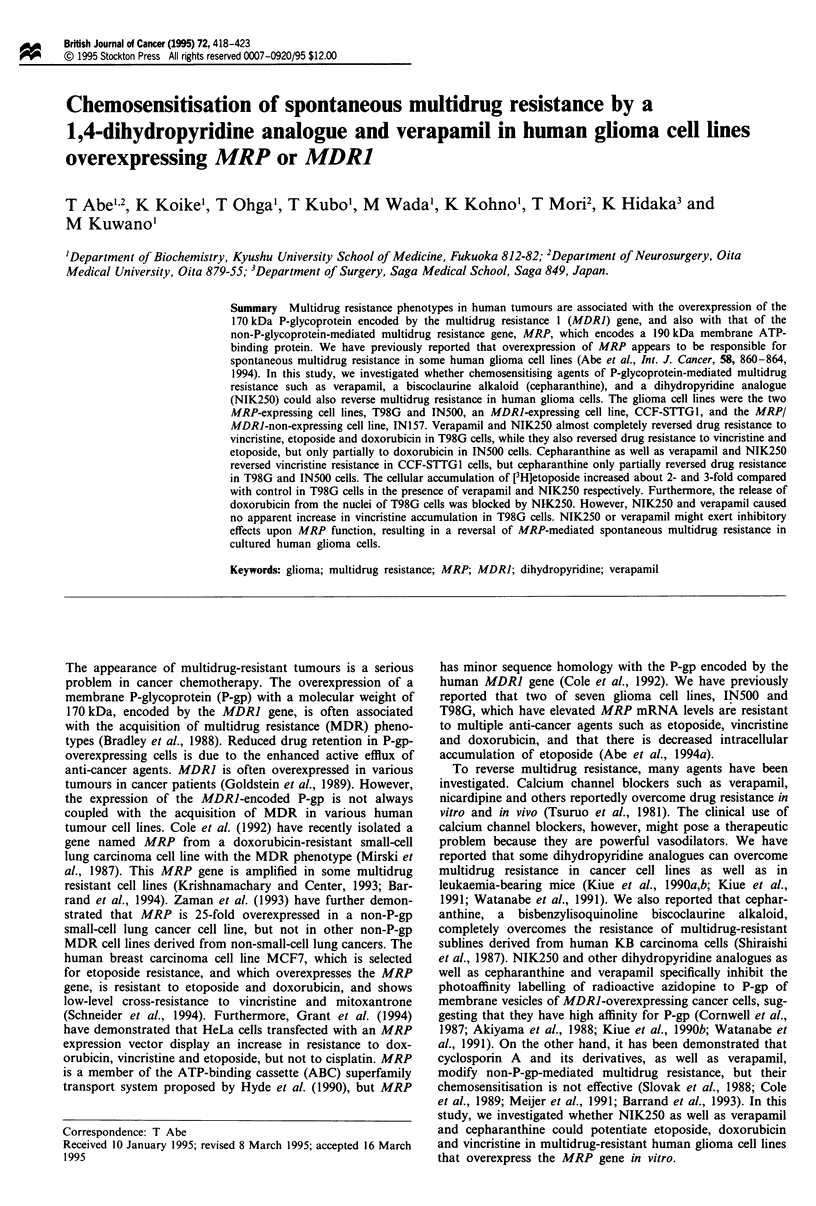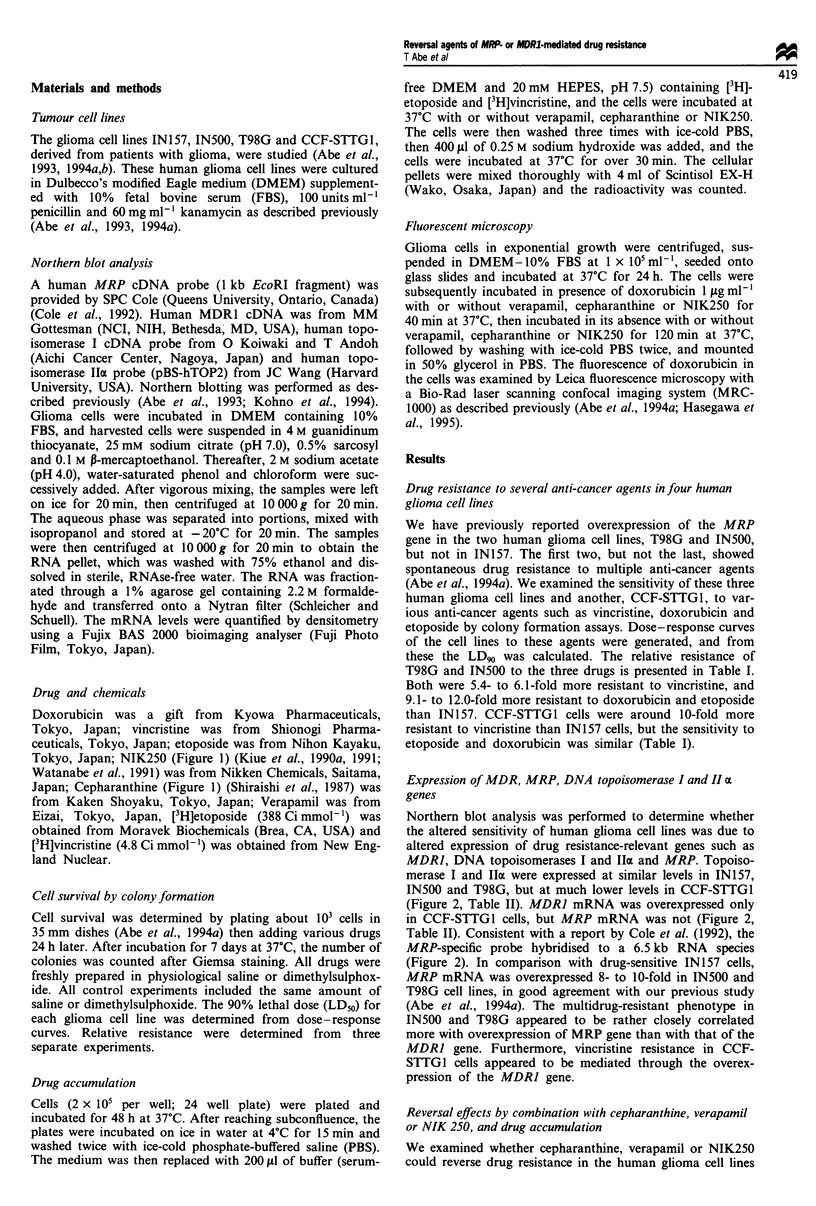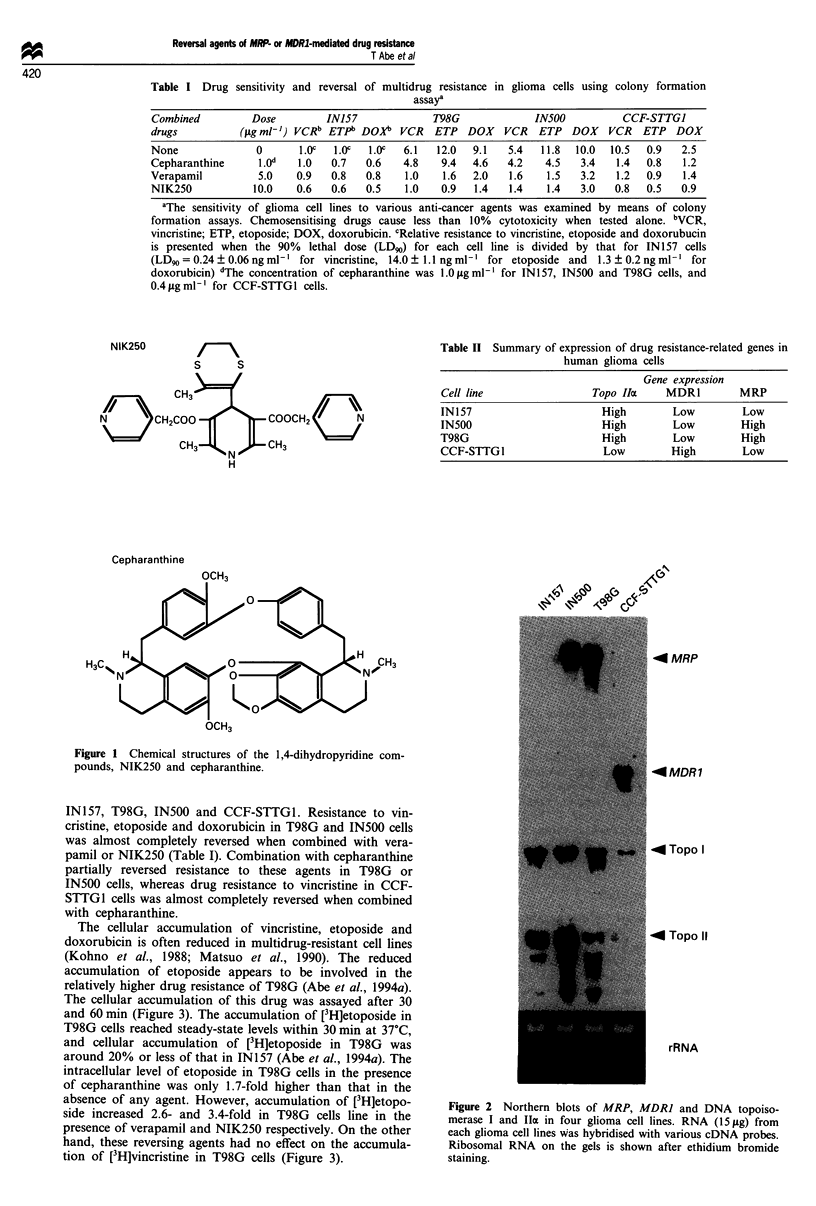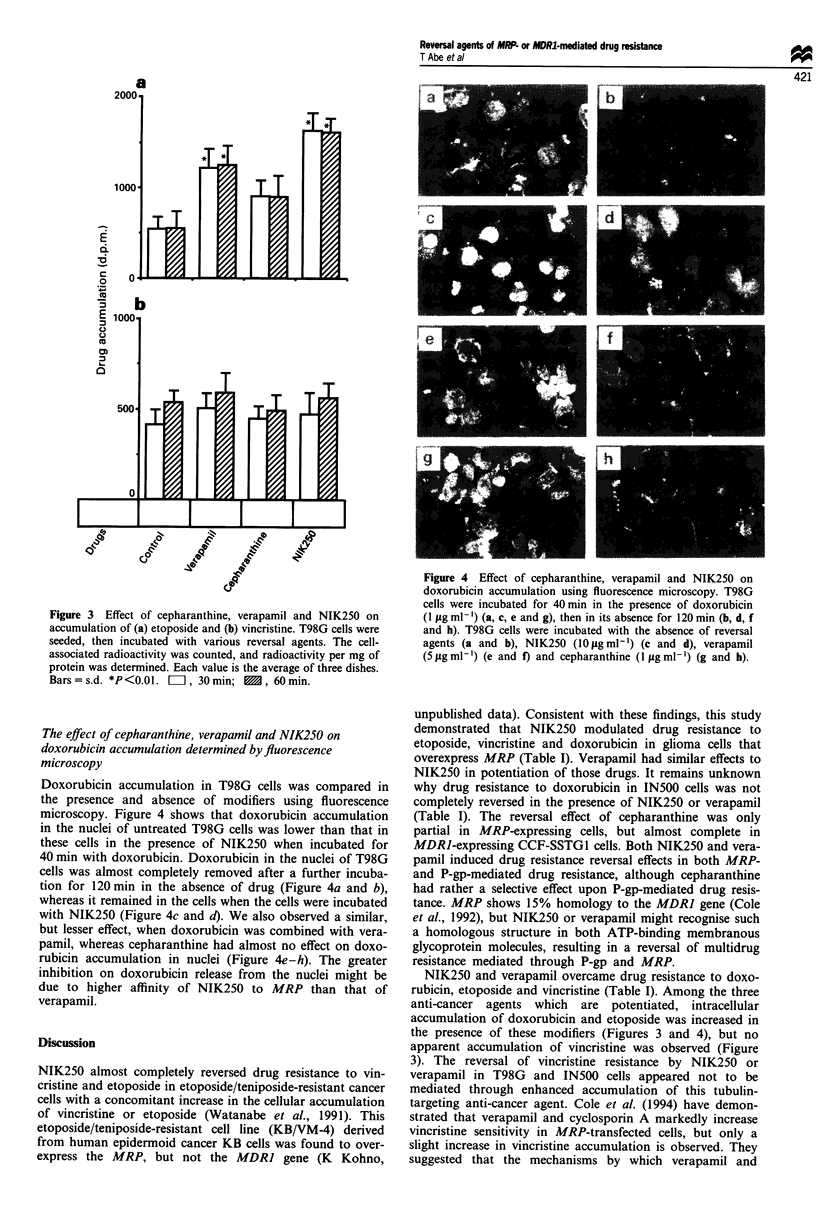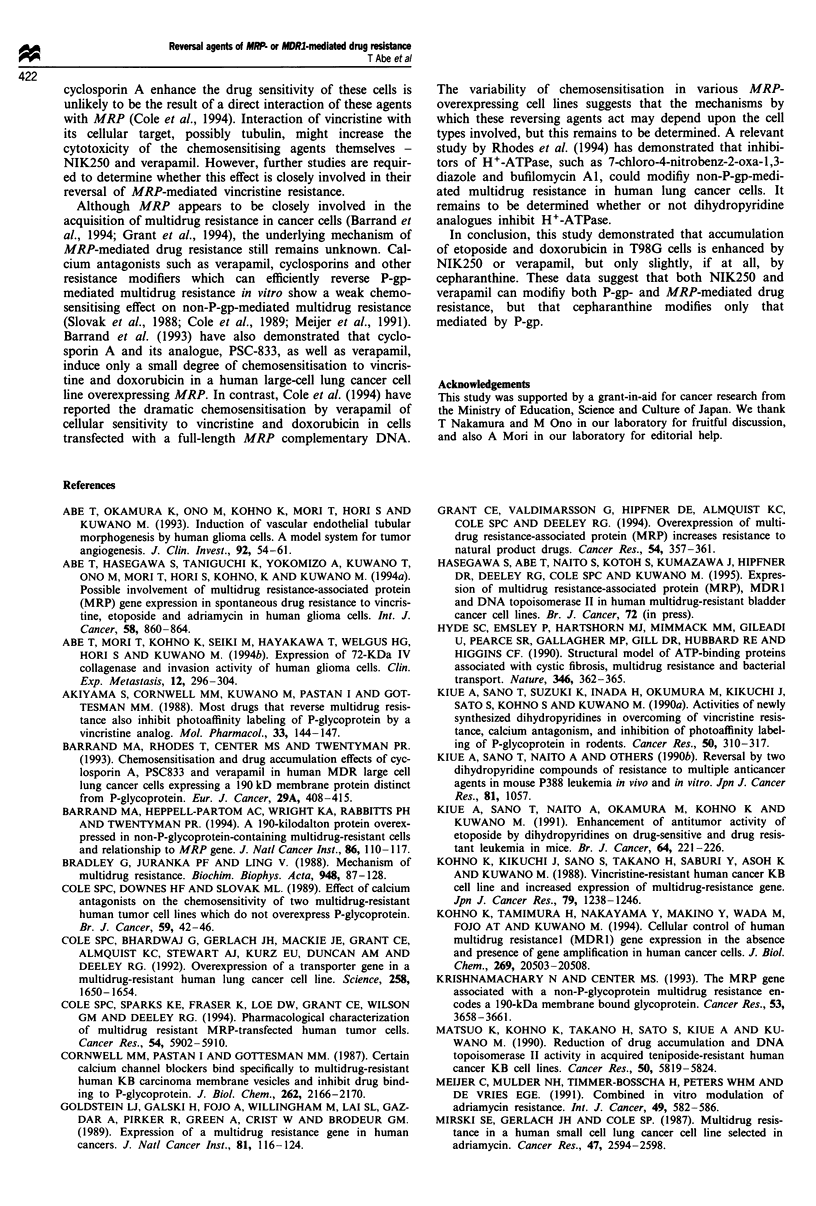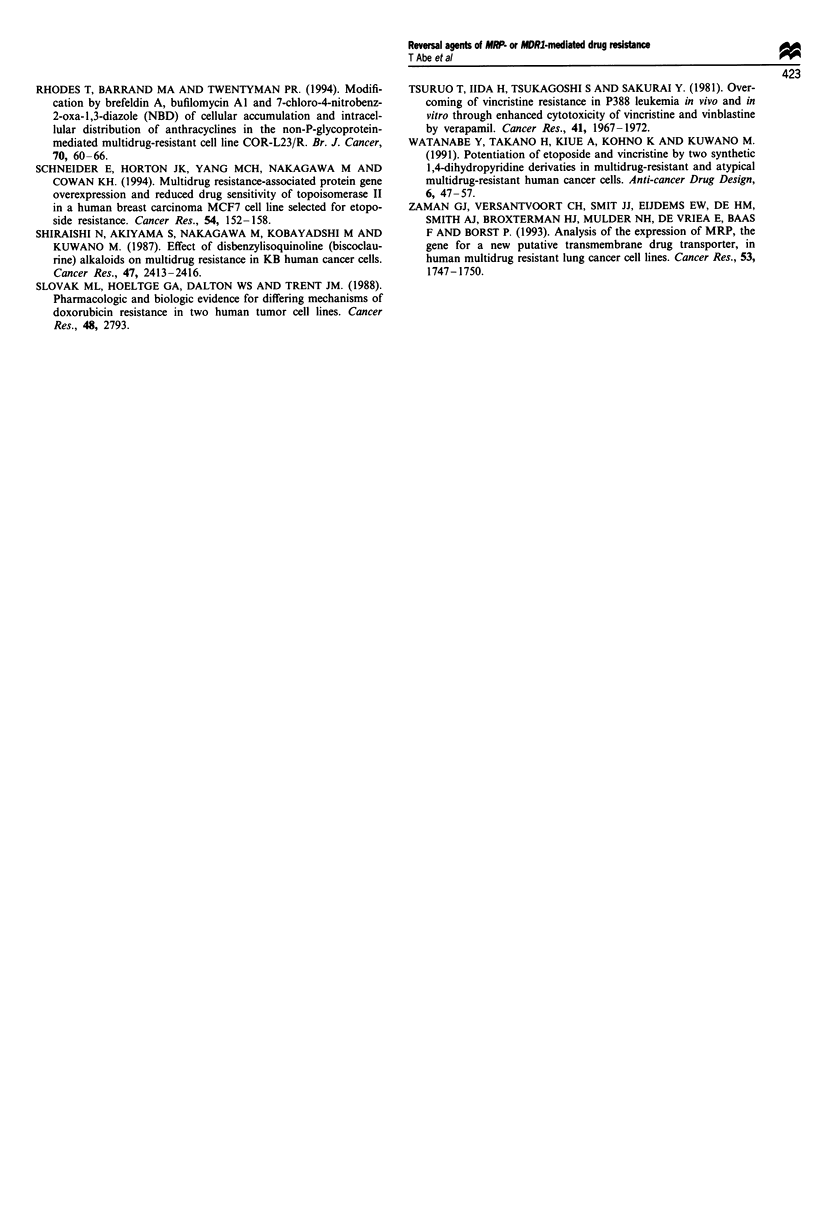# Chemosensitisation of spontaneous multidrug resistance by a 1,4-dihydropyridine analogue and verapamil in human glioma cell lines overexpressing MRP or MDR1.

**DOI:** 10.1038/bjc.1995.348

**Published:** 1995-08

**Authors:** T. Abe, K. Koike, T. Ohga, T. Kubo, M. Wada, K. Kohno, T. Mori, K. Hidaka, M. Kuwano

**Affiliations:** Department of Biochemistry, Kyushu University School of Medicine, Fukuoka, Japan.

## Abstract

**Images:**


British Journal of Cancer (1995) 72, 418-423
© 1995 Stockton Press All rights reserved 0007-0920/95 $12.00

### Chemosensitisation of spontaneous multidrug resistance by a 1,4-dihydropyridine analogue and verapamil in human glioma cell lines overexpressing *MRP* or *MDR1*

T Abe[1,2], K Koike[1], T Ohga[1], T Kubo[1], M Wada[1], K Kohno[1], T Mori[2], K Hidaka[3] and M Kuwano[1]

[1]*Department of Biochemistry, Kyushu University School of Medicine, Fukuoka 812-82;* [2]*Department of Neurosurgery, Oita Medical University, Oita 879-55;* [3]*Department of Surgery, Saga Medical School, Saga 849, Japan.*

**Summary** Multidrug resistance phenotypes in human tumours are associated with the overexpression of the 170 kDa P-glycoprotein encoded by the multidrug resistance 1 (*MDR1*) gene, and also with that of the non-P-glycoprotein-mediated multidrug resistance gene, *MRP*, which encodes a 190 kDa membrane ATP-binding protein. We have previously reported that overexpression of *MRP* appears to be responsible for spontaneous multidrug resistance in some human glioma cell lines (Abe *et al.*, *Int. J. Cancer*, **58**, 860-864, 1994). In this study, we investigated whether chemosensitising agents of P-glycoprotein-mediated multidrug resistance such as verapamil, a biscoclaurine alkaloid (cepharanthine), and a dihydropyridine analogue (NIK250) could also reverse multidrug resistance in human glioma cells. The glioma cell lines were the two *MRP*-expressing cell lines, T98G and IN500, an *MDR1*-expressing cell line, CCF-STTG1, and the *MRP*/*MDR1*-non-expressing cell line, IN157. Verapamil and NIK250 almost completely reversed drug resistance to vincristine, etoposide and doxorubicin in T98G cells, while they also reversed drug resistance to vincristine and etoposide, but only partially to doxorubicin in IN500 cells. Cepharanthine as well as verapamil and NIK250 reversed vincristine resistance in CCF-STTG1 cells, but cepharanthine only partially reversed drug resistance in T98G and IN500 cells. The cellular accumulation of [$^3$H]etoposide increased about 2- and 3-fold compared with control in T98G cells in the presence of verapamil and NIK250 respectively. Furthermore, the release of doxorubicin from the nuclei of T98G cells was blocked by NIK250. However, NIK250 and verapamil caused no apparent increase in vincristine accumulation in T98G cells. NIK250 or verapamil might exert inhibitory effects upon *MRP* function, resulting in a reversal of *MRP*-mediated spontaneous multidrug resistance in cultured human glioma cells.

**Keywords:** glioma; multidrug resistance; *MRP*; *MDR1*; dihydropyridine; verapamil

The appearance of multidrug-resistant tumours is a serious problem in cancer chemotherapy. The overexpression of a membrane P-glycoprotein (P-gp) with a molecular weight of 170 kDa, encoded by the *MDR1* gene, is often associated with the acquisition of multidrug resistance (MDR) phenotypes (Bradley *et al.*, 1988). Reduced drug retention in P-gp-overexpressing cells is due to the enhanced active efflux of anti-cancer agents. *MDR1* is often overexpressed in various tumours in cancer patients (Goldstein *et al.*, 1989). However, the expression of the *MDR1*-encoded P-gp is not always coupled with the acquisition of MDR in various human tumour cell lines. Cole *et al.* (1992) have recently isolated a gene named *MRP* from a doxorubicin-resistant small-cell lung carcinoma cell line with the MDR phenotype (Mirski *et al.*, 1987). This *MRP* gene is amplified in some multidrug resistant cell lines (Krishnamachary and Center, 1993; Barrand *et al.*, 1994). Zaman *et al.* (1993) have further demonstrated that *MRP* is 25-fold overexpressed in a non-P-gp small-cell lung cancer cell line, but not in other non-P-gp MDR cell lines derived from non-small-cell lung cancers. The human breast carcinoma cell line MCF7, which is selected for etoposide resistance, and which overexpresses the *MRP* gene, is resistant to etoposide and doxorubicin, and shows low-level cross-resistance to vincristine and mitoxantrone (Schneider *et al.*, 1994). Furthermore, Grant *et al.* (1994) have demonstrated that HeLa cells transfected with an *MRP* expression vector display an increase in resistance to doxorubicin, vincristine and etoposide, but not to cisplatin. *MRP* is a member of the ATP-binding cassette (ABC) superfamily transport system proposed by Hyde *et al.* (1990), but *MRP* has minor sequence homology with the P-gp encoded by the human *MDR1* gene (Cole *et al.*, 1992). We have previously reported that two of seven glioma cell lines, IN500 and T98G, which have elevated *MRP* mRNA levels are resistant to multiple anti-cancer agents such as etoposide, vincristine and doxorubicin, and that there is decreased intracellular accumulation of etoposide (Abe *et al.*, 1994*a*).

To reverse multidrug resistance, many agents have been investigated. Calcium channel blockers such as verapamil, nicardipine and others reportedly overcome drug resistance *in vitro* and *in vivo* (Tsuruo *et al.*, 1981). The clinical use of calcium channel blockers, however, might pose a therapeutic problem because they are powerful vasodilators. We have reported that some dihydropyridine analogues can overcome multidrug resistance in cancer cell lines as well as in leukaemia-bearing mice (Kiue *et al.*, 1990*a,b*; Kiue *et al.*, 1991; Watanabe *et al.*, 1991). We also reported that cepharanthine, a bisbenzylisoquinoline biscoclaurine alkaloid, completely overcomes the resistance of multidrug-resistant sublines derived from human KB carcinoma cells (Shiraishi *et al.*, 1987). NIK250 and other dihydropyridine analogues as well as cepharanthine and verapamil specifically inhibit the photoaffinity labelling of radioactive azidopine to P-gp of membrane vesicles of *MDR1*-overexpressing cancer cells, suggesting that they have high affinity for P-gp (Cornwell *et al.*, 1987; Akiyama *et al.*, 1988; Kiue *et al.*, 1990*b*; Watanabe *et al.*, 1991). On the other hand, it has been demonstrated that cyclosporin A and its derivatives, as well as verapamil, modify non-P-gp-mediated multidrug resistance, but their chemosensitisation is not effective (Slovak *et al.*, 1988; Cole *et al.*, 1989; Meijer *et al.*, 1991; Barrand *et al.*, 1993). In this study, we investigated whether NIK250 as well as verapamil and cepharanthine could potentiate etoposide, doxorubicin and vincristine in multidrug-resistant human glioma cell lines that overexpress the *MRP* gene *in vitro*.

Correspondence: T Abe
Received 10 January 1995; revised 8 March 1995; accepted 16 March 1995

### Materials and methods

*Tumour cell lines*

The glioma cell lines IN157, IN500, T98G and CCF-STTG1, derived from patients with glioma, were studied (Abe *et al.*, 1993, 1994*a*,*b*). These human glioma cell lines were cultured in Dulbecco's modified Eagle medium (DMEM) supplemented with 10% fetal bovine serum (FBS), 100 units $ml^{-1}$ penicillin and 60 mg $ml^{-1}$ kanamycin as described previously (Abe *et al.*, 1993, 1994*a*).

*Northern blot analysis*

A human *MRP* cDNA probe (1 kb *Eco*RI fragment) was provided by SPC Cole (Queens University, Ontario, Canada) (Cole *et al.*, 1992). Human MDR1 cDNA was from MM Gottesman (NCI, NIH, Bethesda, MD, USA), human topoisomerase I cDNA probe from O Koiwaki and T Andoh (Aichi Cancer Center, Nagoya, Japan) and human topoisomerase IIα probe (pBS-hTOP2) from JC Wang (Harvard University, USA). Northern blotting was performed as described previously (Abe *et al.*, 1993; Kohno *et al.*, 1994). Glioma cells were incubated in DMEM containing 10% FBS, and harvested cells were suspended in 4 M guanidinum thiocyanate, 25 mM sodium citrate (pH 7.0), 0.5% sarcosyl and 0.1 M β-mercaptoethanol. Thereafter, 2 M sodium acetate (pH 4.0), water-saturated phenol and chloroform were successively added. After vigorous mixing, the samples were left on ice for 20 min, then centrifuged at 10 000 *g* for 20 min. The aqueous phase was separated into portions, mixed with isopropanol and stored at −20°C for 20 min. The samples were then centrifuged at 10 000 *g* for 20 min to obtain the RNA pellet, which was washed with 75% ethanol and dissolved in sterile, RNAse-free water. The RNA was fractionated through a 1% agarose gel containing 2.2 M formaldehyde and transferred onto a Nytran filter (Schleicher and Schuell). The mRNA levels were quantified by densitometry using a Fujix BAS 2000 bioimaging analyser (Fuji Photo Film, Tokyo, Japan).

*Drug and chemicals*

Doxorubicin was a gift from Kyowa Pharmaceuticals, Tokyo, Japan; vincristine was from Shionogi Pharmaceuticals, Tokyo, Japan; etoposide was from Nihon Kayaku, Tokyo, Japan; NIK250 (Figure 1) (Kiue *et al.*, 1990*a*, 1991; Watanabe *et al.*, 1991) was from Nikken Chemicals, Saitama, Japan; Cepharanthine (Figure 1) (Shiraishi *et al.*, 1987) was from Kaken Shoyaku, Tokyo, Japan; Verapamil was from Eizai, Tokyo, Japan, [$^3$H]etoposide (388 Ci $mmol^{-1}$) was obtained from Moravek Biochemicals (Brea, CA, USA) and [$^3$H]vincristine (4.8 Ci $mmol^{-1}$) was obtained from New England Nuclear.

*Cell survival by colony formation*

Cell survival was determined by plating about $10^3$ cells in 35 mm dishes (Abe *et al.*, 1994*a*) then adding various drugs 24 h later. After incubation for 7 days at 37°C, the number of colonies was counted after Giemsa staining. All drugs were freshly prepared in physiological saline or dimethylsulphoxide. All control experiments included the same amount of saline or dimethylsulphoxide. The 90% lethal dose ($LD_{50}$) for each glioma cell line was determined from dose–response curves. Relative resistance were determined from three separate experiments.

*Drug accumulation*

Cells ($2 \times 10^5$ per well; 24 well plate) were plated and incubated for 48 h at 37°C. After reaching subconfluence, the plates were incubated on ice in water at 4°C for 15 min and washed twice with ice-cold phosphate-buffered saline (PBS). The medium was then replaced with 200 μl of buffer (serum-free DMEM and 20 mM HEPES, pH 7.5) containing [$^3$H]-etoposide and [$^3$H]vincristine, and the cells were incubated at 37°C with or without verapamil, cepharanthine or NIK250. The cells were then washed three times with ice-cold PBS, then 400 μl of 0.25 M sodium hydroxide was added, and the cells were incubated at 37°C for over 30 min. The cellular pellets were mixed thoroughly with 4 ml of Scintisol EX-H (Wako, Osaka, Japan) and the radioactivity was counted.

*Fluorescent microscopy*

Glioma cells in exponential growth were centrifuged, suspended in DMEM–10% FBS at $1 \times 10^5$ $ml^{-1}$, seeded onto glass slides and incubated at 37°C for 24 h. The cells were subsequently incubated in presence of doxorubicin 1 μg $ml^{-1}$ with or without verapamil, cepharanthine or NIK250 for 40 min at 37°C, then incubated in its absence with or without verapamil, cepharanthine or NIK250 for 120 min at 37°C, followed by washing with ice-cold PBS twice, and mounted in 50% glycerol in PBS. The fluorescence of doxorubicin in the cells was examined by Leica fluorescence microscopy with a Bio-Rad laser scanning confocal imaging system (MRC-1000) as described previously (Abe *et al.*, 1994*a*; Hasegawa *et al.*, 1995).

### Results

*Drug resistance to several anti-cancer agents in four human glioma cell lines*

We have previously reported overexpression of the *MRP* gene in the two human glioma cell lines, T98G and IN500, but not in IN157. The first two, but not the last, showed spontaneous drug resistance to multiple anti-cancer agents (Abe *et al.*, 1994*a*). We examined the sensitivity of these three human glioma cell lines and another, CCF-STTG1, to various anti-cancer agents such as vincristine, doxorubicin and etoposide by colony formation assays. Dose–response curves of the cell lines to these agents were generated, and from these the $LD_{90}$ was calculated. The relative resistance of T98G and IN500 to the three drugs is presented in Table I. Both were 5.4- to 6.1-fold more resistant to vincristine, and 9.1- to 12.0-fold more resistant to doxorubicin and etoposide than IN157. CCF-STTG1 cells were around 10-fold more resistant to vincristine than IN157 cells, but the sensitivity to etoposide and doxorubicin was similar (Table I).

*Expression of MDR, MRP, DNA topoisomerase I and II α genes*

Northern blot analysis was performed to determine whether the altered sensitivity of human glioma cell lines was due to altered expression of drug resistance-relevant genes such as *MDR1*, DNA topoisomerases I and IIα and *MRP*. Topoisomerase I and IIα were expressed at similar levels in IN157, IN500 and T98G, but at much lower levels in CCF-STTG1 (Figure 2, Table II). *MDR1* mRNA was overexpressed only in CCF-STTG1 cells, but *MRP* mRNA was not (Figure 2, Table II). Consistent with a report by Cole *et al.* (1992), the *MRP*-specific probe hybridised to a 6.5 kb RNA species (Figure 2). In comparison with drug-sensitive IN157 cells, *MRP* mRNA was overexpressed 8- to 10-fold in IN500 and T98G cell lines, in good agreement with our previous study (Abe *et al.*, 1994*a*). The multidrug-resistant phenotype in IN500 and T98G appeared to be rather closely correlated more with overexpression of MRP gene than with that of the *MDR1* gene. Furthermore, vincristine resistance in CCF-STTG1 cells appeared to be mediated through the overexpression of the *MDR1* gene.

*Reversal effects by combination with cepharanthine, verapamil or NIK 250, and drug accumulation*

We examined whether cepharanthine, verapamil or NIK250 could reverse drug resistance in the human glioma cell lines

**Table I** Drug sensitivity and reversal of multidrug resistance in glioma cells using colony formation assay[a]

| *Combined drugs* | *Dose* ($\mu g\ ml^{-1}$) | *IN157* *VCR*[b] | *IN157* *ETP*[b] | *IN157* *DOX*[b] | *T98G* *VCR* | *T98G* *ETP* | *T98G* *DOX* | *IN500* *VCR* | *IN500* *ETP* | *IN500* *DOX* | *CCF-STTG1* *VCR* | *CCF-STTG1* *ETP* | *CCF-STTG1* *DOX* |
|---|---|---|---|---|---|---|---|---|---|---|---|---|---|
| None | 0 | 1.0[c] | 1.0[c] | 1.0[c] | 6.1 | 12.0 | 9.1 | 5.4 | 11.8 | 10.0 | 10.5 | 0.9 | 2.5 |
| Cepharanthine | 1.0[d] | 1.0 | 0.7 | 0.6 | 4.8 | 9.4 | 4.6 | 4.2 | 4.5 | 3.4 | 1.4 | 0.8 | 1.2 |
| Verapamil | 5.0 | 0.9 | 0.8 | 0.8 | 1.0 | 1.6 | 2.0 | 1.6 | 1.5 | 3.2 | 1.2 | 0.9 | 1.4 |
| NIK250 | 10.0 | 0.6 | 0.6 | 0.5 | 1.0 | 0.9 | 1.4 | 1.4 | 1.4 | 3.0 | 0.8 | 0.5 | 0.9 |

[a]The sensitivity of glioma cell lines to various anti-cancer agents was examined by means of colony formation assays. Chemosensitising drugs cause less than 10% cytotoxicity when tested alone. [b]VCR, vincristine; ETP, etoposide; DOX, doxorubicin. [c]Relative resistance to vincristine, etoposide and doxorubucin is presented when the 90% lethal dose ($LD_{90}$) for each cell line is divided by that for IN157 cells ($LD_{90} = 0.24 \pm 0.06\ ng\ ml^{-1}$ for vincristine, $14.0 \pm 1.1\ ng\ ml^{-1}$ for etoposide and $1.3 \pm 0.2\ ng\ ml^{-1}$ for doxorubicin) [d]The concentration of cepharanthine was $1.0\ \mu g\ ml^{-1}$ for IN157, IN500 and T98G cells, and $0.4\ \mu g\ ml^{-1}$ for CCF-STTG1 cells.

**Figure 1** Chemical structures of the 1,4-dihydropyridine compounds, NIK250 and cepharanthine.

IN157, T98G, IN500 and CCF-STTG1. Resistance to vincristine, etoposide and doxorubicin in T98G and IN500 cells was almost completely reversed when combined with verapamil or NIK250 (Table I). Combination with cepharanthine partially reversed resistance to these agents in T98G or IN500 cells, whereas drug resistance to vincristine in CCF-STTG1 cells was almost completely reversed when combined with cepharanthine.

The cellular accumulation of vincristine, etoposide and doxorubicin is often reduced in multidrug-resistant cell lines (Kohno *et al.*, 1988; Matsuo *et al.*, 1990). The reduced accumulation of etoposide appears to be involved in the relatively higher drug resistance of T98G (Abe *et al.*, 1994*a*). The cellular accumulation of this drug was assayed after 30 and 60 min (Figure 3). The accumulation of [$^3$H]etoposide in T98G cells reached steady-state levels within 30 min at 37°C, and cellular accumulation of [$^3$H]etoposide in T98G was around 20% or less of that in IN157 (Abe *et al.*, 1994*a*). The intracellular level of etoposide in T98G cells in the presence of cepharanthine was only 1.7-fold higher than that in the absence of any agent. However, accumulation of [$^3$H]etoposide increased 2.6- and 3.4-fold in T98G cells line in the presence of verapamil and NIK250 respectively. On the other hand, these reversing agents had no effect on the accumulation of [$^3$H]vincristine in T98G cells (Figure 3).

**Table II** Summary of expression of drug resistance-related genes in human glioma cells

| *Cell line* | *Gene expression* *Topo IIα* | MDR1 | MRP |
|---|---|---|---|
| IN157 | High | Low | Low |
| IN500 | High | Low | High |
| T98G | High | Low | High |
| CCF-STTG1 | Low | High | Low |

**Figure 2** Northern blots of *MRP*, *MDR1* and DNA topoisomerase I and IIα in four glioma cell lines. RNA ($15\ \mu g$) from each glioma cell lines was hybridised with various cDNA probes. Ribosomal RNA on the gels is shown after ethidium bromide staining.

**Figure 3** Effect of cepharanthine, verapamil and NIK250 on accumulation of (**a**) etoposide and (**b**) vincristine. T98G cells were seeded, then incubated with various reversal agents. The cell-associated radioactivity was counted, and radioactivity per mg of protein was determined. Each value is the average of three dishes. Bars = s.d. $*P<0.01$. ▭, 30 min; ▨, 60 min.

**Figure 4** Effect of cepharanthine, verapamil and NIK250 on doxorubicin accumulation using fluorescence microscopy. T98G cells were incubated for 40 min in the presence of doxorubicin (1 $\mu g\ ml^{-1}$) (**a**, **c**, **e** and **g**), then in its absence for 120 min (**b**, **d**, **f** and **h**). T98G cells were incubated with the absence of reversal agents (**a** and **b**), NIK250 (10 $\mu g\ ml^{-1}$) (**c** and **d**), verapamil (5 $\mu g\ ml^{-1}$) (**e** and **f**) and cepharanthine (1 $\mu g\ ml^{-1}$) (**g** and **h**).

*The effect of cepharanthine, verapamil and NIK250 on doxorubicin accumulation determined by fluorescence microscopy*

Doxorubicin accumulation in T98G cells was compared in the presence and absence of modifiers using fluorescence microscopy. Figure 4 shows that doxorubicin accumulation in the nuclei of untreated T98G cells was lower than that in these cells in the presence of NIK250 when incubated for 40 min with doxorubicin. Doxorubicin in the nuclei of T98G cells was almost completely removed after a further incubation for 120 min in the absence of drug (Figure 4*a* and *b*), whereas it remained in the cells when the cells were incubated with NIK250 (Figure 4*c* and *d*). We also observed a similar, but lesser effect, when doxorubicin was combined with verapamil, whereas cepharanthine had almost no effect on doxorubicin accumulation in nuclei (Figure 4*e*–*h*). The greater inhibition on doxorubicin release from the nuclei might be due to higher affinity of NIK250 to *MRP* than that of verapamil.

### Discussion

NIK250 almost completely reversed drug resistance to vincristine and etoposide in etoposide/teniposide-resistant cancer cells with a concomitant increase in the cellular accumulation of vincristine or etoposide (Watanabe *et al.*, 1991). This etoposide/teniposide-resistant cell line (KB/VM-4) derived from human epidermoid cancer KB cells was found to overexpress the *MRP*, but not the *MDR1* gene (K Kohno, unpublished data). Consistent with these findings, this study demonstrated that NIK250 modulated drug resistance to etoposide, vincristine and doxorubicin in glioma cells that overexpress *MRP* (Table I). Verapamil had similar effects to NIK250 in potentiation of those drugs. It remains unknown why drug resistance to doxorubicin in IN500 cells was not completely reversed in the presence of NIK250 or verapamil (Table I). The reversal effect of cepharanthine was only partial in *MRP*-expressing cells, but almost complete in *MDR1*-expressing CCF-SSTG1 cells. Both NIK250 and verapamil induced drug resistance reversal effects in both *MRP*- and P-gp-mediated drug resistance, although cepharanthine had rather a selective effect upon P-gp-mediated drug resistance. *MRP* shows 15% homology to the *MDR1* gene (Cole *et al.*, 1992), but NIK250 or verapamil might recognise such a homologous structure in both ATP-binding membranous glycoprotein molecules, resulting in a reversal of multidrug resistance mediated through P-gp and *MRP*.

NIK250 and verapamil overcame drug resistance to doxorubicin, etoposide and vincristine (Table I). Among the three anti-cancer agents which are potentiated, intracellular accumulation of doxorubicin and etoposide was increased in the presence of these modifiers (Figures 3 and 4), but no apparent accumulation of vincristine was observed (Figure 3). The reversal of vincristine resistance by NIK250 or verapamil in T98G and IN500 cells appeared not to be mediated through enhanced accumulation of this tubulin-targeting anti-cancer agent. Cole *et al.* (1994) have demonstrated that verapamil and cyclosporin A markedly increase vincristine sensitivity in *MRP*-transfected cells, but only a slight increase in vincristine accumulation is observed. They suggested that the mechanisms by which verapamil and cyclosporin A enhance the drug sensitivity of these cells is unlikely to be the result of a direct interaction of these agents with *MRP* (Cole *et al.*, 1994). Interaction of vincristine with its cellular target, possibly tubulin, might increase the cytotoxicity of the chemosensitising agents themselves – NIK250 and verapamil. However, further studies are required to determine whether this effect is closely involved in their reversal of *MRP*-mediated vincristine resistance.

Although *MRP* appears to be closely involved in the acquisition of multidrug resistance in cancer cells (Barrand *et al.*, 1994; Grant *et al.*, 1994), the underlying mechanism of *MRP*-mediated drug resistance still remains unknown. Calcium antagonists such as verapamil, cyclosporins and other resistance modifiers which can efficiently reverse P-gp-mediated multidrug resistance *in vitro* show a weak chemosensitising effect on non-P-gp-mediated multidrug resistance (Slovak *et al.*, 1988; Cole *et al.*, 1989; Meijer *et al.*, 1991). Barrand *et al.* (1993) have also demonstrated that cyclosporin A and its analogue, PSC-833, as well as verapamil, induce only a small degree of chemosensitisation to vincristine and doxorubicin in a human large-cell lung cancer cell line overexpressing *MRP*. In contrast, Cole *et al.* (1994) have reported the dramatic chemosensitisation by verapamil of cellular sensitivity to vincristine and doxorubicin in cells transfected with a full-length *MRP* complementary DNA. The variability of chemosensitisation in various *MRP*-overexpressing cell lines suggests that the mechanisms by which these reversing agents act may depend upon the cell types involved, but this remains to be determined. A relevant study by Rhodes *et al.* (1994) has demonstrated that inhibitors of $H^+$-ATPase, such as 7-chloro-4-nitrobenz-2-oxa-1,3-diazole and bufilomycin A1, could modifiy non-P-gp-mediated multidrug resistance in human lung cancer cells. It remains to be determined whether or not dihydropyridine analogues inhibit $H^+$-ATPase.

In conclusion, this study demonstrated that accumulation of etoposide and doxorubicin in T98G cells is enhanced by NIK250 or verapamil, but only slightly, if at all, by cepharanthine. These data suggest that both NIK250 and verapamil can modifiy both P-gp- and *MRP*-mediated drug resistance, but that cepharanthine modifies only that mediated by P-gp.

**Acknowledgements**

This study was supported by a grant-in-aid for cancer research from the Ministry of Education, Science and Culture of Japan. We thank T Nakamura and M Ono in our laboratory for fruitful discussion, and also A Mori in our laboratory for editorial help.

**References**

ABE T, OKAMURA K, ONO M, KOHNO K, MORI T, HORI S AND KUWANO M. (1993). Induction of vascular endothelial tubular morphogenesis by human glioma cells. A model system for tumor angiogenesis. *J. Clin. Invest.*, **92**, 54–61.

ABE T, HASEGAWA S, TANIGUCHI K, YOKOMIZO A, KUWANO T, ONO M, MORI T, HORI S, KOHNO, K AND KUWANO M. (1994*a*). Possible involvement of multidrug resistance-associated protein (MRP) gene expression in spontaneous drug resistance to vincristine, etoposide and adriamycin in human glioma cells. *Int. J. Cancer*, **58**, 860–864.

ABE T, MORI T, KOHNO K, SEIKI M, HAYAKAWA T, WELGUS HG, HORI S AND KUWANO M. (1994*b*). Expression of 72-KDa IV collagenase and invasion activity of human glioma cells. *Clin. Exp. Metastasis*, **12**, 296–304.

AKIYAMA S, CORNWELL MM, KUWANO M, PASTAN I AND GOTTESMAN MM. (1988). Most drugs that reverse multidrug resistance also inhibit photoaffinity labeling of P-glycoprotein by a vincristine analog. *Mol. Pharmacol.*, **33**, 144–147.

BARRAND MA, RHODES T, CENTER MS AND TWENTYMAN PR. (1993). Chemosensitisation and drug accumulation effects of cyclosporin A, PSC833 and verapamil in human MDR large cell lung cancer cells expressing a 190 kD membrane protein distinct from P-glycoprotein. *Eur. J. Cancer*, **29A**, 408–415.

BARRAND MA, HEPPELL-PARTOM AC, WRIGHT KA, RABBITTS PH AND TWENTYMAN PR. (1994). A 190-kilodalton protein overexpressed in non-P-glycoprotein-containing multidrug-resistant cells and relationship to *MRP* gene. *J. Natl Cancer Inst.*, **86**, 110–117.

BRADLEY G, JURANKA PF AND LING V. (1988). Mechanism of multidrug resistance. *Biochim. Biophys. Acta*, **948**, 87–128.

COLE SPC, DOWNES HF AND SLOVAK ML. (1989). Effect of calcium antagonists on the chemosensitivity of two multidrug-resistant human tumor cell lines which do not overexpress P-glycoprotein. *Br. J. Cancer*, **59**, 42–46.

COLE SPC, BHARDWAJ G, GERLACH JH, MACKIE JE, GRANT CE, ALMQUIST KC, STEWART AJ, KURZ EU, DUNCAN AM AND DEELEY RG. (1992). Overexpression of a transporter gene in a multidrug-resistant human lung cancer cell line. *Science*, **258**, 1650–1654.

COLE SPC, SPARKS KE, FRASER K, LOE DW, GRANT CE, WILSON GM AND DEELEY RG. (1994). Pharmacological characterization of multidrug resistant MRP-transfected human tumor cells. *Cancer Res.*, **54**, 5902–5910.

CORNWELL MM, PASTAN I AND GOTTESMAN MM. (1987). Certain calcium channel blockers bind specifically to multidrug-resistant human KB carcinoma membrane vesicles and inhibit drug binding to P-glycoprotein. *J. Biol. Chem.*, **262**, 2166–2170.

GOLDSTEIN LJ, GALSKI H, FOJO A, WILLINGHAM M, LAI SL, GAZDAR A, PIRKER R, GREEN A, CRIST W AND BRODEUR GM. (1989). Expression of a multidrug resistance gene in human cancers. *J. Natl Cancer Inst.*, **81**, 116–124.

GRANT CE, VALDIMARSSON G, HIPFNER DE, ALMQUIST KC, COLE SPC AND DEELEY RG. (1994). Overexpression of multidrug resistance-associated protein (MRP) increases resistance to natural product drugs. *Cancer Res.*, **54**, 357–361.

HASEGAWA S, ABE T, NAITO S, KOTOH S, KUMAZAWA J, HIPFNER DR, DEELEY RG, COLE SPC AND KUWANO M. (1995). Expression of multidrug resistance-associated protein (MRP), MDR1 and DNA topoisomerase II in human multidrug-resistant bladder cancer cell lines. *Br. J. Cancer*, **72** (in press).

HYDE SC, EMSLEY P, HARTSHORN MJ, MIMMACK MM, GILEADI U, PEARCE SR, GALLAGHER MP, GILL DR, HUBBARD RE AND HIGGINS CF. (1990). Structural model of ATP-binding proteins associated with cystic fibrosis, multidrug resistance and bacterial transport. *Nature*, **346**, 362–365.

KIUE A, SANO T, SUZUKI K, INADA H, OKUMURA M, KIKUCHI J, SATO S, KOHNO S AND KUWANO M. (1990*a*). Activities of newly synthesized dihydropyridines in overcoming of vincristine resistance, calcium antagonism, and inhibition of photoaffinity labeling of P-glycoprotein in rodents. *Cancer Res.*, **50**, 310–317.

KIUE A, SANO T, NAITO A AND OTHERS (1990*b*). Reversal by two dihydropyridine compounds of resistance to multiple anticancer agents in mouse P388 leukemia *in vivo* and *in vitro*. *Jpn J. Cancer Res.*, **81**, 1057.

KIUE A, SANO T, NAITO A, OKAMURA M, KOHNO K AND KUWANO M. (1991). Enhancement of antitumor activity of etoposide by dihydropyridines on drug-sensitive and drug resistant leukemia in mice. *Br. J. Cancer*, **64**, 221–226.

KOHNO K, KIKUCHI J, SANO S, TAKANO H, SABURI Y, ASOH K AND KUWANO M. (1988). Vincristine-resistant human cancer KB cell line and increased expression of multidrug-resistance gene. *Jpn J. Cancer Res.*, **79**, 1238–1246.

KOHNO K, TAMIMURA H, NAKAYAMA Y, MAKINO Y, WADA M, FOJO AT AND KUWANO M. (1994). Cellular control of human multidrug resistance1 (MDR1) gene expression in the absence and presence of gene amplification in human cancer cells. *J. Biol. Chem.*, **269**, 20503–20508.

KRISHNAMACHARY N AND CENTER MS. (1993). The MRP gene associated with a non-P-glycoprotein multidrug resistance encodes a 190-kDa membrane bound glycoprotein. *Cancer Res.*, **53**, 3658–3661.

MATSUO K, KOHNO K, TAKANO H, SATO S, KIUE A AND KUWANO M. (1990). Reduction of drug accumulation and DNA topoisomerase II activity in acquired teniposide-resistant human cancer KB cell lines. *Cancer Res.*, **50**, 5819–5824.

MEIJER C, MULDER NH, TIMMER-BOSSCHA H, PETERS WHM AND DE VRIES EGE. (1991). Combined in vitro modulation of adriamycin resistance. *Int. J. Cancer*, **49**, 582–586.

MIRSKI SE, GERLACH JH AND COLE SP. (1987). Multidrug resistance in a human small cell lung cancer cell line selected in adriamycin. *Cancer Res.*, **47**, 2594–2598.

RHODES T, BARRAND MA AND TWENTYMAN PR. (1994). Modification by brefeldin A, bufilomycin A1 and 7-chloro-4-nitrobenz-2-oxa-1,3-diazole (NBD) of cellular accumulation and intracellular distribution of anthracyclines in the non-P-glycoprotein-mediated multidrug-resistant cell line COR-L23/R. *Br. J. Cancer*, **70,** 60–66.

SCHNEIDER E, HORTON JK, YANG MCH, NAKAGAWA M AND COWAN KH. (1994). Multidrug resistance-associated protein gene overexpression and reduced drug sensitivity of topoisomerase II in a human breast carcinoma MCF7 cell line selected for etoposide resistance. *Cancer Res.*, **54,** 152–158.

SHIRAISHI N, AKIYAMA S, NAKAGAWA M, KOBAYADSHI M AND KUWANO M. (1987). Effect of disbenzylisoquinoline (biscoclaurine) alkaloids on multidrug resistance in KB human cancer cells. *Cancer Res.*, **47,** 2413–2416.

SLOVAK ML, HOELTGE GA, DALTON WS AND TRENT JM. (1988). Pharmacologic and biologic evidence for differing mechanisms of doxorubicin resistance in two human tumor cell lines. *Cancer Res.*, **48,** 2793.

TSURUO T, IIDA H, TSUKAGOSHI S AND SAKURAI Y. (1981). Overcoming of vincristine resistance in P388 leukemia *in vivo* and *in vitro* through enhanced cytotoxicity of vincristine and vinblastine by verapamil. *Cancer Res.*, **41,** 1967–1972.

WATANABE Y, TAKANO H, KIUE A, KOHNO K AND KUWANO M. (1991). Potentiation of etoposide and vincristine by two synthetic 1,4-dihydropyridine derivaties in multidrug-resistant and atypical multidrug-resistant human cancer cells. *Anti-cancer Drug Design*, **6,** 47–57.

ZAMAN GJ, VERSANTVOORT CH, SMIT JJ, EIJDEMS EW, DE HM, SMITH AJ, BROXTERMAN HJ, MULDER NH, DE VRIEA E, BAAS F AND BORST P. (1993). Analysis of the expression of MRP, the gene for a new putative transmembrane drug transporter, in human multidrug resistant lung cancer cell lines. *Cancer Res.*, **53,** 1747–1750.